# Clinical characteristics and obstetric outcomes of dengue fever in pregnancy: a nationwide population-based study in Taiwan

**DOI:** 10.1186/s12884-026-09199-7

**Published:** 2026-05-02

**Authors:** Meng-Hsuan Chu, Chung-Han Ho, Yi-Chen Chen, Yung-Chieh Tsai, Tian-Ni Kuo, Ing-Luen Shyu

**Affiliations:** 1 Department of Obstetrics and Gynecology, Dalin Tzu Chi Hospital, Chiayi, Taiwan; 2 Department of medical research, Chi Mei medical center, Tainan, Taiwan; 3 Department of Information Management, Southern Taiwan University of Science and Technology, Tainan, Taiwan; 4 Department of Obstetrics and Gynecology, Chi Mei Medical Center, No. 901, Zhonghua Rd., Yongkang Dist., Tainan City, 710 Taiwan; 5 Department of Pharmacy, Chia Nan University of Pharmacy and Science, Tainan, Taiwan

**Keywords:** Dengue fever, Pregnancy, Maternal outcome, Neonatal outcome, Taiwan, Population-based cohort

## Abstract

**Background:**

Dengue fever is a major mosquito-borne viral infection with rising global prevalence in tropical and subtropical regions. Pregnant women represent a particularly vulnerable population due to altered immune and physiological responses. While dengue is endemic in Southeast Asia, population-based data on maternal outcomes in East Asian populations with advanced healthcare systems like Taiwan are scarce.

**Methods:**

A nationwide retrospective descriptive, non-comparative study identified 264 pregnant women with dengue (2012–2018) via the Taiwan NHIRD. Outcomes were analyzed descriptively; comparisons with national statistics are illustrative rather than formal risk estimates.

**Results:**

The median maternal age was 32 years. The most common maternal intervention was Cesarean delivery (68.6%), while ICU admission occurred in 9.1%. For neonatal outcomes, a composite of preterm delivery or incubator care (aggregated due to coding limitations) occurred in 34.9%. Notably, the rate of low birth weight (9.5%) remained comparable to national baselines despite high intervention rates.

**Conclusions:**

High rates of obstetric intervention despite preserved biological outcomes may reflect defensive clinical practices and diagnostic uncertainty during epidemics. Refined guidelines are needed to balance safety and avoid unnecessary surgery.

**Supplementary Information:**

The online version contains supplementary material available at 10.1186/s12884-026-09199-7.

## Introduction

Dengue fever, caused by dengue virus (DENV) and transmitted by Aedes mosquitoes, remains one of the most rapidly spreading mosquito-borne infections worldwide [1–3]. The World Health Organization estimates that approximately 390 million infections occur annually [4], with nearly half of the global population living in at-risk regions. The geographic expansion of Aedes vectors, coupled with urbanization and climate variability, has further intensified the frequency and scale of dengue epidemics in recent decades. In recent decades, Taiwan has experienced recurrent outbreaks, particularly in southern areas such as Kaohsiung and Tainan [5]. Pregnant women constitute a uniquely susceptible group because the physiological, cardiovascular, and immunological adaptations required to maintain fetal tolerance may simultaneously increase vulnerability to infectious diseases. In viral infections, pregnancy-related immune modulation has been associated with altered disease severity and atypical clinical presentations. For dengue infection in particular, endothelial dysfunction, plasma leakage, and thrombocytopenia may be exacerbated during pregnancy, raising concerns regarding hypertensive disorders, hemorrhagic complications, and peripartum management [6–8]. From a clinical perspective, dengue infection during pregnancy presents unique management challenges. Thrombocytopenia and plasma leakage may complicate decisions regarding timing and mode of delivery, anesthesia planning, and peripartum hemorrhage prevention. Moreover, distinguishing dengue-related endothelial dysfunction from pregnancy-specific hypertensive disorders can be clinically difficult, potentially delaying diagnosis or appropriate intervention. Despite these concerns, evidence-based guidance for obstetric management during dengue infection remains limited, particularly in non-endemic settings with intermittent outbreaks.

Several studies from endemic regions, including Brazil, India, and Southeast Asia, have demonstrated associations between maternal dengue infection and adverse outcomes such as preeclampsia, hemorrhage, preterm birth, and perinatal death [9–11]. However, these findings have been inconsistent across settings, and the reported effect sizes vary widely. Such heterogeneity may likely reflect differences in epidemic intensity, access to obstetric care, diagnostic criteria, and study design. A critical distinction exists between known risks in highly endemic regions and settings with intermittent outbreaks and advanced healthcare systems. While studies from endemic regions like Brazil and Southeast Asia have associated maternal dengue with severe outcomes such as preeclampsia and perinatal death, findings remain inconsistent. These variations likely reflect differences in access to obstetric care and diagnostic criteria. In high-resource settings like Taiwan, the primary clinical challenge often involves distinguishing dengue-related endothelial dysfunction from pregnancy-specific hypertensive disorders, such as HELLP syndrome, which can delay diagnosis or trigger defensive interventions. In Taiwan, although dengue fever is a notifiable disease with systematic case reporting, there is limited population-based evidence on its impact during pregnancy [12–14]. Most previous reports have been case series or small observational cohorts [11, 15–19], which are inherently underpowered to evaluate uncommon but clinically significant outcomes. To date, no nationwide population-based study has comprehensively assessed maternal and neonatal risks associated with dengue infection during pregnancy in Taiwan.

Understanding these risks is essential for informing clinical decision-making, antenatal surveillance, and delivery planning during dengue outbreaks. Taiwan’s National Health Insurance Research Database (NHIRD), which covers more than 99% of the population, provides a unique opportunity to address this evidence gap through large-scale, population-based analyses. Given the periodic resurgence of dengue epidemics in southern Taiwan, robust epidemiological data are urgently needed to guide risk stratification and optimize maternal–fetal care in affected regions.

The objective of this study was to provide a nationwide descriptive, non-comparative assessment of maternal and neonatal outcomes among pregnant women diagnosed with dengue fever in Taiwan. Specifically, we aimed to delineate the patterns of obstetric intervention and neonatal surveillance in a high-resource healthcare system, thereby providing a contextual framework for clinical decision-making during future outbreaks .

## Materials and methods

### Study design and data source

**Fig. 1:**
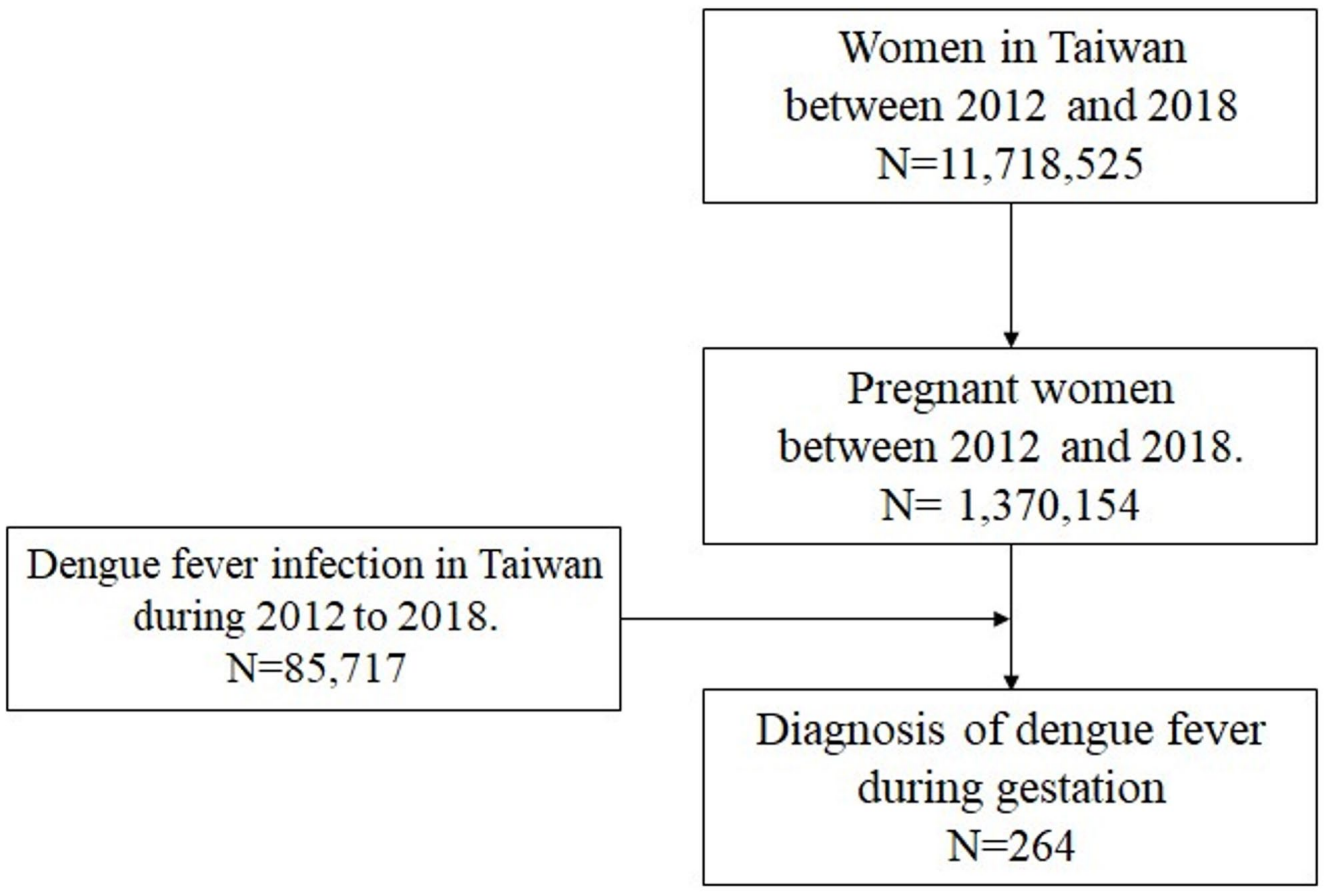
Flow chart of study participant

We conducted a nationwide population-based retrospective descriptive, non-comparative study using data from the Taiwan National Health Insurance Research Database (NHIRD). The National Health Insurance (NHI) program covers more than 99% of the Taiwanese population, and the NHIRD provides comprehensive longitudinal information on demographics, outpatient and inpatient visits, diagnostic codes, and procedure codes. For this study, we extracted claims data from January 2012 to December 2018, identifying all pregnant women who had a diagnosis of dengue fever during gestation. Dengue fever was defined using ICD-9-CM codes 061 and 065 and ICD-10-CM codes A90 and A91. In Taiwan, dengue fever is a notifiable disease requiring laboratory confirmation for official reporting and insurance reimbursement. Therefore, while cases were identified via ICD-9 and ICD-10 codes, these diagnoses are highly likely to have been laboratory-confirmed at the point of care. Only women with a confirmed pregnancy outcome (delivery or miscarriage) were included. The sequential selection process of the study population and the overall research workflow are illustrated in Fig. 1.

### Study population

A total of 264 pregnant women with dengue fever were identified. Maternal baseline characteristics, including age, comorbidities (gestational hypertension, gestational diabetes mellitus, preeclampsia), and parity, were recorded. Maternal age was presented as a continuous variable and also categorized into < 35 years and ≥ 35 years. Geographic distribution of cases was assessed by residence, with the highest proportions reported in Kaohsiung and Tainan, which are areas most affected by dengue epidemics. Given the rarity of dengue infection during pregnancy and its intense seasonal and regional clustering in Taiwan, identifying a matched non-dengue control group that remains comparable in terms of both temporal epidemic strain and clinical context was methodologically difficult. Matching could potentially introduce selection bias regarding healthcare access during outbreaks. Therefore, this study was designed as a noncomparing descriptive nationwide analysis, utilizing aggregate national health statistics from the same period as a contextual backdrop to illustrate clinical trends. No non-dengue control group was matched; outcomes are interpreted in the context of aggregate national statistics.

### Outcome measures

Maternal and neonatal outcomes were defined according to ICD-9-CM, ICD-10-CM, and NHI procedure codes (Supplementary Table 1). Maternal adverse outcomes included Cesarean section, postpartum hemorrhage, obstetric complications (e.g., uterine rupture, sepsis), length of hospital stay, and intensive care unit (ICU) admission. Neonatal adverse outcomes included preterm delivery or requirement for neonatal incubator care (< 37 weeks or requiring incubator care), low birth weight (< 2,500 g), respiratory distress syndrome, neonatal ICU admission, stillbirth, and neonatal dengue or bacterial sepsis. Due to coding limitations within the NHIRD, preterm delivery and neonatal incubator care were aggregated, as specific administrative codes for incubator use often overlap with preterm care billing in Taiwan. These definitions were harmonized across ICD-9 and ICD-10 codes to ensure comparability across years. Clinical severity was characterized not only by diagnostic codes but also by intensity of care. Given the potential for inconsistent application of severity-specific codes (A90 vs. A91) in administrative data, ICU admission was pre-specified as the primary proxy indicator for maternal clinical severity to ensure a more objective evaluation of morbidity. Regarding neonatal dengue, it is important to clarify that administrative claims data do not provide the clinical or laboratory granularity required to distinguish between true vertical (in utero) transmission and early postnatal exposure. Therefore, cases identified by these codes are classified as potential neonatal exposures.

### Statistical analysis

Descriptive statistics were used to summarize baseline characteristics and outcome distributions. Given the rarity of dengue infection during pregnancy and the lack of temporally and clinically comparable non-dengue controls within the same epidemic context, this study was designed as a descriptive nationwide analysis rather than a comparative risk estimation study. Categorical variables were expressed as frequencies and percentages, and continuous variables were reported as medians with interquartile ranges (IQRs). Aggregate national health statistics from the same period were utilized solely as a contextual backdrop to illustrate clinical trends. No formal matching or inferential risk calculations were performed between the study cohort and the national baseline. Given the lack of a matched non-dengue comparison group in the current dataset, inferential analyses such as regression modeling were not performed. Instead, the proportion of maternal and neonatal outcomes was reported and contextualized with findings from prior studies conducted in other dengue-endemic countries. Statistical analyses were performed using SAS version 9.4 (SAS Institute, Cary, NC, USA).

The study protocol was approved by the Institutional Review Board of Chi Mei Medical Center (IRB No. 11309-010). The requirement for informed consent was waived by the IRB because the study utilized de-identified administrative data from the NHIRD, ensuring the protection of participants’ privacy.

## Results

**Fig. 2:**
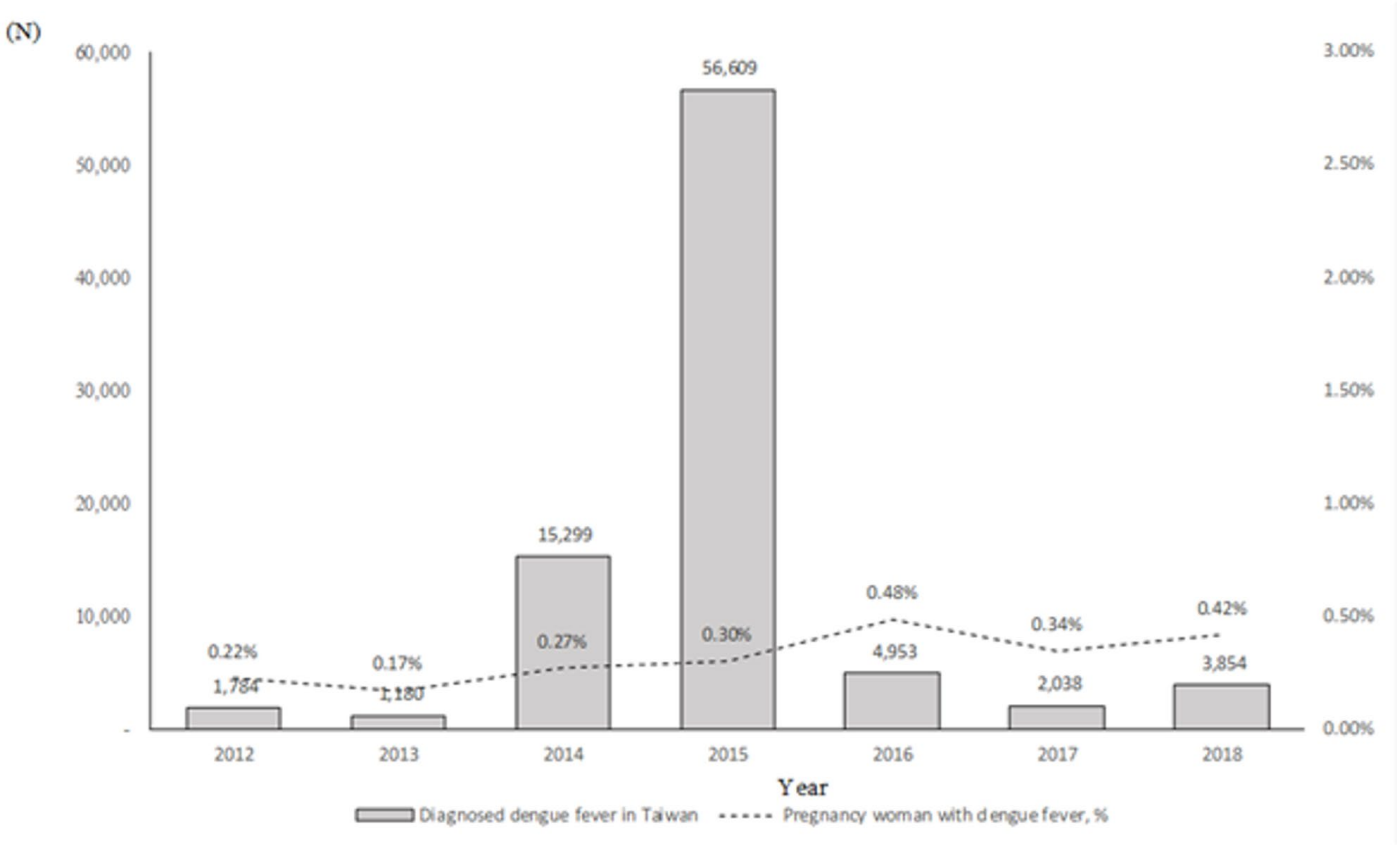
Temporal distribution of maternal dengue infection in Taiwan (2012–2018). The bars represent the annual number of confirmed dengue cases nationwide, while the curve indicates the number of dengue-infected pregnant women during the same period. The prominent peaks in 2014 and 2015 directly correspond to the two largest nationwide dengue outbreaks in Taiwan’s recent history, during which the healthcare system in southern Taiwan faced significant strain

The study flowchart (Fig. 1) depicts the identification of the 264 pregnant women with confirmed dengue fever included in this nationwide cohort. From the initial screening of the NHIRD database between 2012 and 2018, participants were filtered based on confirmed diagnosis codes and documented pregnancy outcomes. A total of 264 pregnant women diagnosed with dengue fever were identified from the Taiwan National Health Insurance Research Database between 2012 and 2018. As shown in Fig. 2, cases were highly concentrated during 2014 and 2015, which represented a ten-fold increase compared to non-epidemic years within the study period.

The median maternal age was 32 years (interquartile range [IQR], 28–34 years). Of these women, 198 (75%) were younger than 35 years, and 66 (25.0%) were aged 35 years or older. Pregnancy-related comorbidities were relatively uncommon: gestational hypertension was observed in 4 women (1.5%), gestational diabetes mellitus in 28 women (10.6%), and preeclampsia in 8 women (3.0%). Only one woman (0.4%) presented with signs of miscarriage during the course of dengue infection. Nearly all pregnancies were singleton (98.1%), indicating that the adverse outcomes reported in this study were unlikely to be confounded by multiple gestations. In terms of geographic distribution, 130 women (49.2%) resided in Kaohsiung, 92 (34.9%) in Tainan, and the remaining 42 (15.9%) in other regions of Taiwan. This spatial clustering may reflect the concentration of dengue outbreaks in southern Taiwan during the study period (Table 1). Due to the mandatory nature of the National Health Insurance system, there were no missing data for maternal demographics or documented pregnancy outcomes.

**Table 1:** Pregnant women with dengue fever (*n* = 264)

	*N*	%
Maternal age		
Median (Q1-Q3)	32 (28–34)	
< 35	198	75.0
≧ 35	66	25.0
GHTN	4	1.52
GDM	28	10.61
Pre-eclampsia	8	3.03
Singleton	259	98.11
Area		
Kaohsiung	130	49.24
Tainan	92	34.85
Others	42	15.91

All categorical data are presented as *N* (number of events), % (percentage). A*bbreviations*: *GHTN* gestational hypertension, *GDM* gestational diabetes mellitus

As shown in Table 2, adverse maternal outcomes were common among dengue-infected pregnancies. Cesarean delivery was the most frequent intervention, performed in 181 women, accounting for 68.6% of all deliveries. Postpartum hemorrhage occurred in 22 women (8.3%), and 62 women (23.5%) experienced other obstetric complications, including uterine rupture, sepsis, or abnormal labor patterns. The median length of hospital stay was 3 days (IQR, 3–5 days), with some women requiring prolonged hospitalization due to complications. Importantly, 24 women (9.1%) required admission to the intensive care unit (ICU), reflecting the severity of maternal illness in this cohort. These findings are consistent with the observation that dengue fever during pregnancy was associated with high rates of obstetric interventions and critical care utilization. Regarding disease severity as defined by ICD coding, the vast majority of cases in this cohort were identified as dengue fever (A90), with cases explicitly coded as severe dengue (A91) being relatively infrequent. To provide a more robust assessment of maternal morbidity in this high-resource setting, we utilized ICU admission rates (9.1%) as a reliable clinical proxy for severe disease manifestations.

**Table 2:** Maternal and fetal adverse outcomes

Outcome Variables	No. of Events (*N* = 264)	Proportion (%)	95% CI	National Baseline (%)*[20]
Maternal Outcomes				
Cesarean Section	181	68.6	63.0–74.2	38.4
Postpartum Hemorrhage	22	8.3	5.0–11.7	-
Hospital stay, day Median (Q1-Q3)	3 (3–5)	-	-	-
ICU Admission	24	9.1	5.6–12.6	-
Neonatal Outcomes				
Preterm Birth (< 37 weeks) or needed baby incubator	92	34.9	29.1–40.7	10.88
Low Birth Weight (< 2500 g)	25	9.5	6.0–13.0	10.94
Neonatal ICU admission	12	4.55	-	-
Dengue fever or bacterial sepsis	5	1.89	0.6–4.4	-

*Abbreviations*: *ICU* intensive care unit, *CI* confidence interval. Proportion rates reflect the proportion of cases within the identified cohort of 264 pregnant women

* National baseline data are provided for illustrative purposes and contextual comparison only

Adverse neonatal outcomes were also frequent. A composite neonatal outcome—defined as either preterm delivery (< 37 weeks of gestation) or admission for neonatal incubator care—was observed in 92 cases (34.9%). Low birth weight (< 2,500 g) was observed in 25 newborns (9.5%). Nine neonates (3.4%) developed respiratory distress syndrome, and 12 (4.6%) required admission to the neonatal ICU for further management. Neonatal infectious complications were notable: five infants (1.9%) were diagnosed with dengue fever or bacterial sepsis shortly after birth, raising questions regarding potential neonatal exposure, which may encompass both vertical transmission and early postnatal infection. Compared to the national baseline, the dengue cohort demonstrated a markedly higher incidence of Cesarean sections and perterm births (68.6% vs. 38.4% nationally) [20]. However, the rate of low birth weight (9.5%) remained comparable to the national average of approximately 6–13% reported during the same period. Overall, these results demonstrate a significant burden of neonatal morbidity associated with maternal dengue infection (Table 2).

The proportion of dengue fever among pregnant women varied across the study years and was closely aligned with nationwide dengue epidemics. As shown in Fig. 2, cases were highly concentrated during 2014 and 2015, which corresponded to the largest dengue outbreaks in Taiwan in the past decade. Seasonal variation was also apparent, with the majority of cases occurring during the summer and early autumn months, paralleling the seasonal proliferation of Aedes mosquitoes. Regional clustering was evident, with Kaohsiung and Tainan consistently representing the epicenters of maternal dengue infections, mirroring the broader community transmission patterns in Taiwan.

## Discussion

The dissociation between intensive clinical intervention and limited evidence of severe fetal growth impairment is consistent with the finding that obstetric and neonatal management during dengue outbreaks in Taiwan is strongly influenced by healthcare system responses. While the Cesarean section rate in our cohort was nearly double the national average, it is important to interpret this comparison as a descriptive reflection of clinical practice during epidemics rather than a controlled risk assessment. It should be noted that the composite neonatal outcome (preterm delivery or incubator care) reflects healthcare utilization patterns rather than intrinsic neonatal pathology alone. In the context of Taiwan’s healthcare system, incubator admission often serves as a precautionary measure for monitoring potential infection, which may inflate the perceived morbidity.

The most striking finding of this study is the Cesarean section rate approaching 70%, which is nearly double the national average in Taiwan [20]. Importantly, this elevated rate should not be interpreted as evidence that Cesarean delivery is physiologically safer in the setting of dengue-associated thrombocytopenia. On the contrary, existing evidence and guidelines from dengue-endemic regions consistently recommend vaginal delivery whenever possible, as surgical delivery exposes larger tissue surfaces and vascular beds, thereby increasing the risk of uncontrolled hemorrhage in coagulopathic patients [15, 21]. However, a significant limitation remains: administrative claims data do not provide granular, indication-level information for each Cesarean delivery. While clinical triggers are not granularly captured in administrative data, the observed dissociation is striking: the 68.6% Cesarean rate occurred alongside a low birth weight rate (9.5%) and stillbirth rate (0.4%) that are comparable to national averages. If acute biological fetal compromise (e.g., severe fetal distress or placental insufficiency) were the primary drivers of these interventions, a concomitant increase in adverse neonatal biological outcomes would be expected. The preservation of these outcomes further supports the hypothesis that many interventions were likely defensive or precautionary in nature.

We propose that the markedly elevated Cesarean rate observed in this cohort may reflect a combination of diagnostic ambiguity and risk-containment strategies within a high-resource healthcare system. Severe dengue infection shares substantial clinical and laboratory overlap with pregnancy-specific hypertensive disorders, particularly pre-eclampsia and HELLP syndrome. Thrombocytopenia, elevated liver enzymes, endothelial dysfunction, plasma leakage, ascites, and pleural effusions may be present in both conditions [13]. Furthermore, pregnancy-related hemodilution may mask the hemoconcentration typically used to identify plasma leakage in dengue, further complicating differentiation during the acute phase of illness [22, 23]. The rapid onset of plasma leakage in dengue can mimic the endothelial dysfunction seen in HELLP, making it difficult to definitively exclude the latter during the acute phase. This diagnostic uncertainty often compels clinicians to pursue early delivery as a risk-containment strategy.

In epidemic settings, this diagnostic “fog of war” may prompt clinicians to favor early delivery as a means of stabilizing maternal condition or preventing further deterioration, especially when HELLP syndrome cannot be confidently excluded. Within Taiwan’s medico-legal environment—where baseline Cesarean rates are already high and malpractice concerns are prominent [24]—Cesarean delivery may be perceived as a more controlled and defensible intervention, despite its lack of physiological advantage in dengue-associated coagulopathy. Thus, the high Cesarean rate observed in this study is consistent with defensive clinical decision-making patterns reported in epidemic settings [25].

Another key finding of this study is the pronounced discrepancy between the high proportion of the composite neonatal outcome (preterm delivery or incubator care, 34.9%) and the relatively low rate of low birth weight (9.5%), which closely approximates national background levels. In standard obstetric populations, preterm birth and low birth weight are highly correlated; therefore, the observed dissociation are strongly consistent with the observation that a substantial proportion of neonates classified as “preterm” or requiring incubator care were likely late-preterm or term infants with normal birth weights. Furthermore, our study is subject to the potential for misclassification inherent in administrative claims data, where severity-specific ICD codes (e.g., A90 vs. A91) may be used inconsistently by clinicians. To ensure a more objective evaluation of morbidity, we utilized ICU admission rates (9.1%) as a primary proxy for clinical severity, as procedure-based billing for intensive care is less prone to diagnostic coding bias .

This pattern is best interpreted as an artifact of healthcare utilization and surveillance intensity rather than intrinsic neonatal morbidity. Due to limitations of administrative claims data, preterm delivery diagnoses may include cases of threatened preterm labor that ultimately resulted in near-term or term birth. Similarly, neonatal incubator admission in Taiwan’s National Health Insurance system encompasses not only prematurity but also precautionary observation, isolation, or monitoring for potential vertical transmission during infectious outbreaks. During dengue epidemics, neonatologists may adopt a low threshold for incubator admission to facilitate close observation for fever, thrombocytopenia, or early signs of infection, even among asymptomatic term infants.

Taken together, these findings suggest with that dengue infection during pregnancy in Taiwan triggers heightened neonatal surveillance rather than widespread biological compromise of fetal growth. The preserved low birth weight rate supports the conclusion that, in this cohort, dengue infection did not result in substantial intrauterine growth restriction at the population level.

The majority of cases in this study occurred during the large dengue outbreaks of 2014–2015 and were geographically concentrated in southern Taiwan, regions that experienced substantial healthcare system strain during these epidemics. Under such conditions, clinicians may reasonably adopt more conservative and intervention-heavy management strategies, including early delivery, intensive maternal monitoring, and precautionary neonatal observation. These patterns may reflect a healthcare system prioritizing risk containment in the face of clinical uncertainty rather than a direct manifestation of viral pathogenicity.

While our data showed a small number of neonates diagnosed with dengue shortly after birth, these findings cannot be interpreted as definitive proof of vertical transmission. As highlighted in recent literature, distinguishing between transplacental transmission and exposure in the immediate peripartum period remains a significant diagnostic challenge in epidemic settings [26]. Given the limitations of administrative data, these instances are best characterized as potential exposures. The identification of these cases emphasizes the need for high clinical suspicion and routine screening of neonates born to infected mothers, even in the absence of obvious maternal severity, to ensure early detection of potentially severe neonatal complications. This diagnostic uncertainty further reinforces the clinical practice of precautionary neonatal monitoring observed in our cohort. These findings have significant implications for clinical training and the adaptation of obstetric guidelines in Taiwan. There is a clear need for formalized diagnostic pathways that integrate serological testing for dengue with standard hypertensive workups to reduce unnecessary surgical interventions. Clinical education should focus on the ‘dissociation’ identified in this study, encouraging a shift toward conservative management and vaginal delivery in stable patients to mitigate the risks associated with major surgery in the presence of coagulopathy.

This interpretation has important implications for future guideline development in non-endemic or intermittently endemic regions. Improved diagnostic tools to distinguish dengue infection from pregnancy-specific hypertensive disorders, clearer criteria for timing and mode of delivery, and standardized neonatal monitoring protocols may help reduce unnecessary surgical intervention while maintaining maternal and neonatal safety. To formally validate the defensive-medicine hypothesis in the context of maternal dengue, future prospective research is warranted. Such studies should ideally incorporate real-time clinical decision-making interviews and document diagnostic challenges at the point of care. Capturing the dynamic clinical reasoning of obstetricians during active outbreaks would provide the evidence needed to distinguish between biological necessity and risk-averse management strategies.

This study is subject to limitations inherent in administrative claims data, including potential misclassification of diagnoses and limited availability of granular clinical information such as platelet counts, dengue severity, specific obstetric indications (e.g., fetal monitoring strips), and the exact trimester of infection, and the specific gestational age at delivery. The composite definition of preterm delivery and incubator care may overestimate true biological prematurity. Additionally, the absence of a matched non-dengue comparison group precludes causal inference. Furthermore, there is the inherent limitations of administrative claims data, including potential coding bias or misclassification between dengue fever and severe dengue. To mitigate this limitation, we prioritized objective clinical outcomes, such as ICU admission, which more accurately reflect the actual clinical burden and severity of the disease in the studied population. Although we have acknowledged the lack of exact gestational weeks, it is important to note that the NHIRD does not provide specific trimester data for outpatient visits. Consequently, the potential differential risks across trimesters could not be fully explored. We suggest that future longitudinal studies incorporate clinical EMR data to refine risk stratification and provide more tailored clinical recommendations. Nevertheless, as a nationwide descriptive analysis, this study provides valuable insight into real-world obstetric and neonatal practice patterns during dengue epidemics in a high-resource healthcare setting.

## Conclusion

This nationwide population-based study demonstrates that dengue fever during pregnancy in Taiwan is associated with high rates of obstetric and neonatal intervention. Notably, these interventions occurred despite severe biological outcomes remaining relatively uncommon. The dissociation between intensive medical intervention and preserved fetal growth outcomes suggests that epidemic-related diagnostic uncertainty and healthcare system responses play a substantial role in shaping perinatal management. These findings highlight the importance of refining diagnostic differentiation between dengue infection and pregnancy-specific complications, as well as developing context-specific obstetric guidelines to safely balance maternal–fetal protection with avoidance of unnecessary surgical intervention during dengue outbreaks.

## Supplementary Information

Supplementary Material 1. Supplement Table 1. Definitions of maternal and fetal outcomes using ICD-9-CM/ICD-10-CM diagnosis codes and NHI procedure codes.

## Data Availability

The data that support the findings of this study are available from the Health and Welfare Data Science Center (HWDC) in Taiwan, but restrictions apply to the availability of these data, which were used under license for the current study, and are not publicly available. Data are however available from the authors upon reasonable request and with permission of the Ministry of Health and Welfare, Taiwan.
