# Supplementary material for: Clinical characteristics and obstetric outcomes of dengue fever in pregnancy: a nationwide population-based study in Taiwan
Source: BMC Pregnancy Childbirth. 2026 May 2;26:668. doi: 10.1186/s12884-026-09199-7 (PMC13285017; doi:10.1186/s12884-026-09199-7)
Supplement: Supplementary file 1 — Supplementary Material 1. Supplement Table 1. Definitions of maternal and fetal outcomes using ICD-9-CM/ICD-10-CM diagnosis codes and NHI procedure codes. [file 12884_2026_9199_MOESM1_ESM.docx]

**Supplement Table 1**. Definitions of maternal and fetal outcomes using ICD-9-CM/ICD-10-CM diagnosis codes and NHI procedure codes.

| **Maternal** | **ICD-10-CM code** | **ICD-9-CM code** | **Procedure code** |
| --- | --- | --- | --- |
| Postpartum hemorrhage | O67/O72.0/O72.1/O72.2 | 666(666.0-666.3)/667(667.0-667.1) | 55015C/ 81005C/ 81029C |
| Caesarean section | by NHI procedure codes only | | 81004C/81011C |
| GHTN | O13 | 642.0/642.1/642.2/642.3/642.9 |  |
| Pre-eclampsia | O14 | 642.4/642.5/642.6/642.7 | 55016C |
| GDM | O24.4 | 648.0 |  |
| ICU | by NHI procedure codes only | | 02011K/02012A/02013B |
| Complicated by intrapartum | O69/O70/O71/O73/O74/O75/O85/O86/O88/O90 | 646(646.0-646.9) |  |
| Signs of a miscarriage | O03 | 634(634.0-634.9) |  |
| **Fetal** |  |  |  |
| Low birth weight, <2,500 g | P07.1 | V21.3 |  |
| Preterm delivery, <37 weeks or needed baby incubator | O60 | 644(644.0-644.2) | 57118B/57119B |
| Respiratory distress syndrome of newborn | P22 | 768/769/770 |  |
| ICU | by NHI procedure codes only | | 02011K/02012A/02013B |
| Lack of expected normal physiological development in childhood | R62 | 764.0/764.1/764.2/764.9 |  |
| Dengue fever or bacterial sepsis | A90/A91/P36/P37 | 771 |  |
